# Do strategically demanding sports shape hand function alike? Distinct profiles of dexterity and grip strength in basketball and badminton athletes

**DOI:** 10.3389/fphys.2026.1940944

**Published:** 2026-09-30

**Authors:** Xinpeng Gao, Jinlong Hou, Chengzhe Du, Yanan Gao, Fei Kong, Ming Li

**Affiliations:** 1 School of Physical Education, Yulin University, Yulin, China; 2 School of Physical Education, Liaocheng University, Liaocheng, China; 3 School of Physical Education, Huaibei Normal University, Huaibei, China; 4 College of Education and Sports Sciences, Yangtze University, Jingzhou, China

**Keywords:** badminton, basketball, handgrip strength, manual dexterity, perceptual–cognitive motor, strategic sports

## Abstract

**Purpose:**

Long-term participation in strategic sports may enhance hand dexterity and cognitive function, but sport-specific differences in manual control, force production, and cognition remain unclear. This study compared hand dexterity, grip strength, and cognitive performance among basketball athletes, badminton athletes, and non-athletes.

**Methods:**

This cross-sectional observational study included 90 right-handed participants (6 females, 84 males; mean age 20.57 ± 1.64 years) divided into the basketball athlete group (BAG), badminton athlete group (BmAG), and non-athlete controls (NCG). Participants completed the Purdue Pegboard Test (PPT), Grooved Pegboard Test (GPT), handgrip strength test (HD), Montreal Cognitive Assessment (MoCA), and Stroop Color–Word Test (SCWT). Results: Both BAG and BmAG outperformed NCG in PPT dominant-hand scores, combined task scores, GPT completion speed, MoCA scores, and SCWT word reading (all P < 0.05). BmAG also showed superior PPT non-dominant-hand performance, bimanual coordination, and total scores compared with NCG, and better bimanual coordination than BAG (BmAG vs. NCG: P < 0.001, d = 0.96; BmAG vs. BAG: P = 0.033, d = 0.66). BAG demonstrated greater dominant-hand grip strength than NCG (P = 0.007, d = 0.90), whereas BmAG did not. No group differences were found in SCWT inhibitory control or cognitive flexibility.

**Conclusion:**

Badminton participation was associated with superior fine motor control and bimanual coordination, whereas basketball participation was associated with greater dominant-hand grip strength. Both athletic groups showed better selected manual and cognitive outcomes than NCG. However, the cross-sectional, male-skewed sample cannot establish causality, and the MoCA findings should be interpreted cautiously because of its ceiling effect in healthy young participants. Longitudinal or intervention studies are warranted. After Holm–Bonferroni correction for multiple outcomes, the group effects for PPT non-dominant-hand performance, dominant-hand grip strength, and Stroop word reading did not remain significant and are therefore interpreted as exploratory.

## Introduction

The hand is one of the most versatile and frequently used structures of the upper limb. It is essential for fine motor control and daily activities requiring manual skills and plays an important role in sport-specific performance. Hand function develops through precise neuromuscular regulation and is further shaped by long-term, sport-specific training. Abilities such as manual dexterity, visuomotor coordination, and bimanual coordination are important components of motor performance and share common neural substrates with higher-order cognitive processes, including executive function, attentional control, and working memory. Accordingly, hand motor performance may be associated with cognitive functioning (Chen et al., 2022 — a visuomotor-integration study of elite badminton players; Tan et al., 2016 — a comparison of cortical networks between athletes and novices). Previous studies have suggested that participation in badminton and basketball may be associated with sport-specific adaptations in hand function (King et al., 2024; Patti et al., 2025). Badminton athletes have been reported to demonstrate greater grip endurance and faster reaction times than non-athletes (King et al., 2024), whereas long-term basketball training may reduce interlimb asymmetries in hand strength and dexterity (Akpinar, 2016; Patti et al., 2025), although the former evidence was obtained in female basketball players and the latter in school-age children. Nevertheless, evidence from systematically controlled studies comparing how different strategically demanding sports influence hand function and its cognitive correlates remains limited.

Beyond their effects on hand function, sport-specific demands and perception–action coupling may also contribute to the development of executive function in athletes (Akpinar, 2016). Long-term specialized training can induce task-specific adaptations in motor control, interlimb coordination, and neural regulation, and sport experience has been consistently associated with superior hand dexterity (Akpinar, 2016; King et al., 2024; Patti et al., 2025; Tan et al., 2016; Vaivre-Douret et al., 2021). According to the principle of specificity, the extent to which training transfers to a specific motor ability depends on the movement structure, force-production characteristics, and neural control requirements of the task. Training stimuli that closely resemble the demands of a sport are therefore more likely to enhance the corresponding motor abilities (Formenti et al., 2021). From a kinetic-chain perspective, hand actions are not isolated distal movements but result from the coordinated generation and transmission of force across the trunk, upper limb, and hand. Thus, effective manual dexterity may depend partly on the efficient integration of these body segments (Vaivre-Douret et al., 2021; Young, 2006).

Basketball and badminton are both strategically demanding sports in which athletes must continuously interpret environmental information, make rapid decisions, and adjust their movements in real time. These perceptual and cognitive requirements may strengthen the neural regulation of fine manual control and contribute to an integrated perception–cognition–action system (Farrow and Abernethy, 2003; Wei et al., 2024). Farrow and Abernethy emphasized the importance of perception–action coupling in movement control and decision-making during strategic sports (Farrow and Abernethy, 2003). Similarly, neuroscientific evidence suggests that neural adaptations associated with long-term participation in highly strategic skill training may improve the efficiency of distal motor execution (Wei et al., 2024). However, most previous studies have examined hand dexterity from a single perspective, such as training adaptation, neural mechanisms, or biomechanics. Few studies have investigated these factors within an integrated framework that simultaneously considers sport specificity, perception–action coupling, and cognitive engagement.

Empirical evidence directly comparing athletes from sports with different strategic demands remains limited. Previous studies have generally focused on a single sport or have broadly compared athletes with non-athletes, whereas relatively few have examined differences among athletes participating in distinct types of strategically demanding sports (Kioumourtzoglou et al., 1998; Qi et al., 2022). Although basketball and badminton both involve substantial strategic and perceptual demands, they differ considerably in their requirements for manual control. Basketball is a high-intensity, contact-based sport that places considerable demands on dynamic spatial judgment, rapid decision-making, and bilateral limb coordination (Barnsley and Rabinovitch, 1970; Schmidt et al., 2019). In contrast, badminton is a fast-paced net sport that relies more heavily on rapid reactions, visual tracking, and precise distal control of the fingers and hand (Kioumourtzoglou et al., 1998; Xiao et al., 2025). These differences in strategic demands, force-production patterns, and modes of hand use may contribute to distinct profiles of hand dexterity among athletes from the two sports. However, direct comparative evidence addressing this issue remains scarce.

Against this background, the present study compared basketball athletes, badminton athletes, and non-athlete controls using a battery of standardized assessments, including the Purdue Pegboard Test, Grooved Pegboard Test, handgrip strength test, Montreal Cognitive Assessment, and Stroop Color–Word Test. The study aimed to determine whether participation in different strategically demanding sports is associated with distinct patterns of hand dexterity and to explore the potential contributions of sport-specific demands, perception–action coupling, and cognitive engagement to these differences. We hypothesized that, compared with non-athletes, both basketball and badminton athletes would demonstrate superior manual dexterity on pegboard tasks and greater handgrip strength. We further hypothesized that both athlete groups would exhibit better cognitive performance, reflecting the shared demands of strategically demanding sports for rapid information processing, attentional control, and response inhibition.

## Materials and methods

### Participants

This study employed a cross-sectional observational design; participants were not randomized to sport groups, and no pre-training baseline assessments were available. Participants were recruited in China from 23 January to 28 February 2026. Recruitment was conducted through targeted telephone communication with coaches of provincial basketball and badminton sports clubs and with teachers of a nearby university (from which the non-athlete controls were drawn), followed by preliminary eligibility screening. Ninety individuals were ultimately enrolled. Before participation, all participants provided written informed consent. The study protocol complied with the Declaration of Helsinki and was approved by the Ethics Committee of the College of Education and Sports Sciences, Yangtze University (Approval No. CESS-20260123003; approved 23 January 2026). Eligibility requirements were an age of 18–22 years, a BMI of 18.5–23.9 kg/m², and stable dietary and sleeping habits during the preceding 3 months, including 7–9 h of sleep per night and no prolonged dieting or binge eating. Participants were also required to have no congenital or chronic disease, recent sports injury, diagnosed psychiatric or neurological disorder, or long-term use of medication that could affect physical or cognitive function. In addition, they had to be nonsmokers, have normal or corrected-to-normal vision, and be cleared for exercise according to the Physical Activity Readiness Questionnaire (PAR-Q). In total, 152 individuals were contacted (61 basketball athletes, 57 badminton athletes, and 34 potential controls); 24 were excluded at telephone screening (primarily for BMI outside the eligible range, recent injury, or insufficient training history) and 12 declined participation. Ninety participants (30 per group) were then enrolled in order of screening until each group reached its target; the remaining eligible volunteers were not enrolled because allocation was already complete. The athletes’ competitive levels were verified through official registration records held by provincial or national sports associations, as well as records of participation in formal leagues, to ensure they met provincial or national competitive standards. Within each sports group, participants were selected from multiple teams affiliated with clubs in the same region (four basketball teams and five badminton teams) and received standardised guidance from the same professional coaching team (comprising coaches and fitness trainers). This design standardised the training environment whilst effectively controlling for clustering bias that might arise from a single team. Furthermore, candidates’ dietary and sleep habits were assessed via a structured self-administered questionnaire during the eligibility interview, covering habitual sleep duration, sleep regularity and overall dietary patterns over the past three months; any candidates who did not meet the aforementioned lifestyle criteria were excluded at the screening stage.

The athletes in both sport-specific groups had achieved provincial- or national-level competitive status and had participated in formal league competitions. All had completed at least 6 months of systematic intensive training before enrollment. The basketball athlete group (BAG) had a mean training history of 9.8 ± 2.2 years and trained for 3.5 ± 1.1 h per day on average. The corresponding values for the badminton athlete group (BmAG) were 9.2 ± 2.3 years and 3.4 ± 1.1 h per day, respectively. The non-athlete control group (NCG) consisted of individuals who engaged in less than 30 min of moderate-intensity physical activity per week and had not regularly participated in organized sport or structured exercise during the preceding 3 years. Participants were excluded if they had a diagnosed neurological disorder or learning disability, an upper- or lower-limb injury within the previous 6 months that could influence test performance, cognitive impairment, or any other physical condition likely to affect the assessment outcomes. Before the formal experiment, participants completed a questionnaire covering demographic and training information, including age, sport specialty, years of continuous training, medical history, and hand dominance. Eligibility for physical activity was subsequently reconfirmed using the PAR-Q. The required sample size was estimated *a priori* using G*Power version 3.1.9.2 (F-test for a fixed-effects one-way ANOVA; α = 0.05; power (1 − β) = 0.80; number of groups = 3). The prespecified primary outcome was the bimanual Purdue Pegboard score. The effect size (f = 0.35) was derived from a pilot study of 30 volunteers (10 per group, drawn from the same recruitment sources), in which a one-way ANOVA of the pilot bimanual scores yielded f = 0.35. The analysis indicated that at least 84 participants were needed. Accordingly, 90 participants were recruited to enhance statistical power and minimize the influence of potential attrition. An independent-samples t test confirmed that the two athlete groups did not differ significantly in training years (t(58) = 1.03, P = 0.306) or daily training hours (t(58) = 0.35, P = 0.727). The three groups did not differ significantly in age (F(2,87) = 1.03, P = 0.361), body mass (F(2,87) = 2.26, P = 0.111), or BMI (F(2,87) = 0.42, P = 0.660) (**Table 1**). Height differed across groups (F(2,87) = 10.39, P < 0.001): the basketball athletes were taller than both the NCG and the badminton athletes (both P < 0.001), whereas the latter two groups did not differ in height (P > 0.05). The sample comprised 84 men and 6 women, distributed evenly across groups (NCG: 2 women, 28 men; BAG: 2 women, 28 men; BmAG: 2 women, 28 men); the small number of women per group precluded meaningful sex-stratified or sex-adjusted analyses.

**Table 1:** Baseline characteristics of the three participant groups.

Variable	NCG (n=30)	BAG (n=30)	BmAG (n=30)	F(2,87)	P
Age (years)	20.51 ± 2.04	20.90 ± 1.78	20.30 ± 0.86	1.03	0.361
Height (m)	1.77 ± 0.05	1.82 ± 0.05	1.76 ± 0.06	10.39	<0.001
Body mass (kg)	71.23 ± 8.90	74.57 ± 10.67	69.27 ± 9.62	2.26	0.111
BMI (kg/m²)	22.78 ± 2.67	22.54 ± 2.73	22.18 ± 2.35	0.42	0.660

Data are presented as mean ± SD. Between-group differences were analyzed by one-way ANOVA. NCG, non-athlete control group; BAG, basketball athlete group; BmAG, badminton athlete group. Height differed significantly (P < 0.001): the basketball athletes were taller than both other groups; all remaining variables did not differ significantly across groups.

### Experimental procedures

All data were collected between 10:00 and 16:00 to minimize potential circadian effects. Participants attended two laboratory visits 48 h apart. The first visit involved familiarization with the laboratory environment and testing procedures, measurement of height and body mass, and an explanation of the study protocol and associated risks. During the second visit, participants completed five assessments in a randomized and counterbalanced order: the Grooved Pegboard Test and Purdue Pegboard Test for hand dexterity, the Montreal Cognitive Assessment and Stroop Color–Word Test for cognitive function, and a handgrip strength test using a dynamometer. A standardized practice trial preceded each assessment, and the testing session lasted approximately 40 min. All assessments were conducted in a provincial-level laboratory located adjacent to the sports club. To minimize variability associated with training background and environmental conditions, participants in the BAG and BmAG were recruited from the same sports club and training environment. Participants in the NCG were recruited from a nearby university. The control participants were drawn from the same geographical area and met the same general eligibility criteria as the athlete groups. Stable dietary and sleeping habits were verified only through the structured self-report screening items described above; no objective (e.g., actigraphic or diary-based) verification was performed, so quantitative similarity between groups in these characteristics could not be established. In addition, because the NCG was recruited from a nearby university rather than from the sports clubs, residual group differences in unmeasured lifestyle characteristics (e.g., daily physical activity, sleep, and nutrition) cannot be excluded.

### Hand dynamometer

Handgrip strength was assessed using a Jamar 5030J1 hydraulic dynamometer. Testing posture followed the recommendations of the American Society of Hand Therapists: participants were seated with the shoulder adducted and neutrally rotated, the elbow flexed at 90°, the forearm in a neutral position, and the wrist positioned in 0°–30° of dorsiflexion (Fess and Moran, 1981). Three maximal isometric contractions were performed with each hand, and each contraction was maintained for 6 s. A 60-s rest interval was provided between trials to minimize muscular fatigue. The final grip strength value for each hand was calculated as the mean of the three trials. This averaging procedure has demonstrated better test–retest reliability than either a single trial or the highest value of three trials in healthy adults; a recent study using the GripAble device (36 participants, repeated testing separated by approximately 2 weeks) reported the highest reliability and precision when using the mean of three trials, with ICC = 0.97 and MDC% = 12.9 for the dominant hand, and ICC = 0.94 and MDC% = 18.2 for the non-dominant hand (Kudin et al., 2025).

### Grooved pegboard test

Manual dexterity was measured with the Grooved Pegboard Test according to a standardized administration procedure (Merker and Podell, 2011). Participants were first familiarized with the task and then completed the assessment with the designated hand. The objective was to place 25 uniquely grooved pegs into the corresponding openings of a 5 × 5 pegboard as quickly as possible. Right-handed participants began in the upper-left position and worked across the board from left to right, progressing downward row by row. Left-handed participants followed the reverse horizontal direction, beginning in the upper-right position. Before a peg could be inserted, its groove had to be correctly oriented to match the slot. One practice trial was provided, followed by three recorded trials. The examiner started the stopwatch with the verbal command “start” and stopped it when the final peg was inserted. The completion time, in seconds, was used as the raw performance score (Wang et al., 2024). The final score for each hand is defined as the average of the three recorded trials. During the test, the dominant hand is always tested first, followed by the non-dominant hand, with the hands tested alternately; there is a 30-second rest interval between consecutive trials and between the two hands, and the testing order is the same for all participants.

### Purdue pegboard test

Fine motor performance was evaluated using the PPT, a standardized measure of manual and finger dexterity (Chen et al., 2024; McLellan, 2011). Testing was performed in a quiet laboratory environment. The apparatus consisted of a pegboard with two columns of 25 holes and separate compartments for pins, washers, and collars. Pins were stored in the lateral compartments, whereas washers and collars were positioned in the central compartments. The assessment included four timed tasks: dominant-hand pin placement for 30 s, non-dominant-hand pin placement for 30 s, simultaneous pin placement with both hands for 30 s, and a 60-s assembly task. During the assembly task, participants alternately used their left and right hands to construct units in the following order: pin–washer–collar–washer. The height of the table and chair and the participant’s seated posture were kept consistent across all testing sessions. Participants completed standardized practice trials before the recorded assessment. A 15-s rest period was provided between consecutive subtests, and a 1-min rest was given after each complete battery. The four-subtest battery was performed three times, with the entire procedure lasting approximately 12 min. For the first three tasks, performance was quantified by the number of pins correctly placed within the allotted time; for the assembly task, the total number of correctly placed components (pins, washers, and collars) was recorded, with each complete pin–washer–collar–washer sequence corresponding to four points. The mean of the three trials was calculated for each subtest. The examiner monitored task execution and timing throughout testing, while participants could request clarification or a brief pause when necessary to ensure safe and valid assessment. The dominant + non-dominant + bimanual (“combined”) score is a deterministic sum of the three preceding subtests and is reported as a summary index rather than as an independent outcome. Although the four-subtest battery was repeated three times within approximately 12 min, the three administrations used a fixed order and identical rest intervals for all participants, and all group comparisons were based on trial-averaged scores; any uniform practice or fatigue effect would therefore not be expected to bias between-group differences, and participants were permitted additional rest on request.

### Stroop color–word test

To account for the potential effect of cognition on performance in the GPT (Tolle et al., 2020), cognitive function was assessed immediately after the GPT using a Stroop paradigm adapted from Periáñez et al (Periáñez et al., 2021). The protocol comprised three timed conditions. In the Stroop Word-Reading (SWR) condition, the words “red,” “green,” and “blue” were printed in black and displayed in randomly ordered vertical columns. Participants read the words aloud, with consecutive repetitions within a column avoided. In the Stroop Color-Naming (SCN) condition, stimuli consisting of four different strings were presented in red, green, or blue ink, and participants named the ink color. The stimulus sequence was arranged to prevent immediate repetition of the color or the word order used in the SWR condition. In the Stroop Color–Word (SCW) condition, the written word and ink color were incongruent. Participants were instructed to disregard the word meaning and identify only the ink color to induce the Stroop interference effect. Each condition lasted 45 s. The examiner recorded the number of correct responses completed during each condition, and this value was used as the task score. Higher scores indicated better cognitive performance (Wang et al., 2024). The task was self-paced: stimuli were printed in vertical columns, and participants progressed through the columns as quickly as possible, so the number of items attempted varied between participants within the fixed 45-s window. The three conditions were administered in a fixed order (SWR, SCN, SCW) for all participants, consistent with the standardized protocol of Periáñez et al. (2021) and with the requirement to avoid immediate stimulus repetitions across conditions.

### Montreal cognitive assessment

The MoCA was included among the five assessments completed during the second laboratory visit. The order of testing was randomized and counterbalanced across participants. Once the general familiarization procedures had been completed and the participant’s readiness had been confirmed, the MoCA was administered individually by trained assessors using the validated Chinese paper-based version and standardized instructions (Chen et al., 2016). No specific practice trial was conducted for the MoCA. The assessment required approximately 10 min. Before administration, the examiner confirmed that appropriate visual and auditory conditions were available, and participants were not permitted to use assistive devices. Testing was carried out in a quiet, private room at the provincial laboratory near the club. Responses were scored immediately after completion and independently verified by a second researcher. The original data were entered into the study database on the day of testing and stored without personal identifiers. If discomfort occurred, participants were permitted to take a short break, which was documented to support procedural consistency and data quality.

### Statistical analysis

Data analyses were conducted with SPSS 27.0 (IBM, Armonk, NY, USA) and GraphPad Prism 9.0.0 (GraphPad Software, San Diego, CA, USA). The results generated by the two programs were cross-checked for consistency: all analyses were first performed in SPSS 27.0 and independently repeated in GraphPad Prism 9.0.0; any discrepancy would have prompted a joint re-check of the data file, syntax, and outputs by two analysts, with the SPSS results treated as primary. No discrepancies were identified. Continuous variables are expressed as mean ± standard deviation (SD). The Shapiro–Wilk test was used to evaluate distributional normality, whereas Levene’s test was applied to examine the equality of variances. Levene’s test was evaluated at α = 0.05; when P < 0.05, equal variances were rejected and Welch’s ANOVA was used with Games–Howell *post hoc* comparisons. When the assumptions of normality and homoscedasticity were satisfied, differences among groups were examined using a one-way analysis of variance (ANOVA). When equal variances could not be assumed, Welch’s ANOVA was used. For significant omnibus effects, Tukey’s test was selected for homogeneous variances, and the Games–Howell procedure was used when variances were unequal. The magnitude of the ANOVA effect was expressed as eta squared (η²), with values interpreted according to Cohen’s conventions (Cohen, 1988) as trivial (< 0.01), small (0.01–0.06), moderate (0.06–0.14), or large (> 0.14). Pairwise differences were accompanied by Cohen’s d, interpreted as small (|d| < 0.20), moderate (0.20 ≤ |d| < 0.80), or large (|d| ≥ 0.80). All tests were two-sided, and statistical significance was set at P < 0.05. Relevant test statistics and effect sizes were reported to assist interpretation of both statistical and practical significance. To limit inflation of the family-wise error rate, the outcomes were classified *a priori* into a primary outcome (bimanual Purdue Pegboard score) and secondary outcomes (the remaining PPT subtests, GPT completion times, handgrip strength, MoCA, and SCWT measures), and the Holm–Bonferroni procedure was applied across the 16 outcome-level omnibus tests. After Holm correction, the omnibus effects for the PPT dominant-hand, bimanual, combined, and assembly scores, both GPT completion times, and the MoCA remained statistically significant, whereas the effects for the PPT non-dominant-hand score, dominant-hand grip strength, and SWR did not remain significant and are therefore interpreted as exploratory. Under the more conservative Bonferroni threshold (α/16 ≈ 0.003), the combined PPT score effect (P = 0.005) would not remain significant. The P values reported in **Tables 2**–**4** and **Figure 1** are uncorrected. The reported one-way and Welch’s ANOVAs are unadjusted for potential confounders.

**Table 2:** Purdue pegboard test.

Purdue pegboard test	NCG (n=30)	BAG (n=30)	BmAG (n=30)	Overall F	P/η²
Dominant Hand Scoring	14.76 ± 1.89	15.86 ± 1.54*	16.43 ± 1.56**	7.432	0.001/0.146
Non-dominant Hand Scoring	13.76 ± 1.90	14.43 ± 1.87	14.96 ± 1.72*	3.107	0.049/0.067
Both Hand Scoring	11.56 ± 1.47	11.96 ± 1.62#	13.00 ± 1.52**	6.661	0.002/0.133
D+N+Both Hand Scoring	40.10 ± 5.27	42.26 ± 2.53	44.40 ± 4.81**	5.738	0.005/0.117
Assembly Scoring	32.30 ± 7.36	38.30 ± 4.30**	39.10 ± 4.70**	12.610	<0.001/0.225

Inter-group differences were analyzed by one-way ANOVA with post hoc Tukey test. NCG, Non-athlete control group; BAG, Basketball athletes group; BmAG, Badminton athletes group. *P < 0.05, **P < 0.01 vs. NCG; # P < 0.05 vs. BmAG; no symbol indicates P > 0.05.

**Table 3:** Grooved pegboard test and handgrip strength.

Parameter	NCG (n=30)	BAG (n=30)	BmAG (n=30)	Overall F	P/η²
GPT PlacingTime Dominant Hand (s)	59.51 ± 3.14	53.92 ± 1.87**	54.22 ± 5.90**	14.730	<0.001/0.291
GPT Placing Time Nondominant Hand (s)	65.86 ± 2.58	60.32 ± 2.71**	61.16 ± 8.64**	8.740	<0.001/0.167
Handgrip Strength Dominant Hand (kg)	41.53 ± 7.24	48.08 ± 7.31*	44.18 ± 9.61	4.831	0.010/0.100
Handgrip Strength Nondominant Hand (kg)	38.32 ± 6.81	43.05 ± 7.23	39.31 ± 8.88	3.040	0.053/0.065

Inter-group differences were analyzed by one-way ANOVA with post hoc Tukey test. GPT, Grooved Pegboard Test; NCG, Non-athlete control group; BAG, Basketball athletes group; BmAG, Badminton athletes group. *P < 0.05, **P < 0.01 vs. NCG; no symbol indicates P > 0.05. For variables with unequal variances (both GPT completion times and dominant-hand grip strength), Welch’s ANOVA with Games–Howell post hoc tests was used; the remaining variables were analyzed by standard one-way ANOVA with Tukey’s HSD. No comparison between the two athlete groups (BAG vs. BmAG) reached statistical significance in this table.

**Figure 1:**
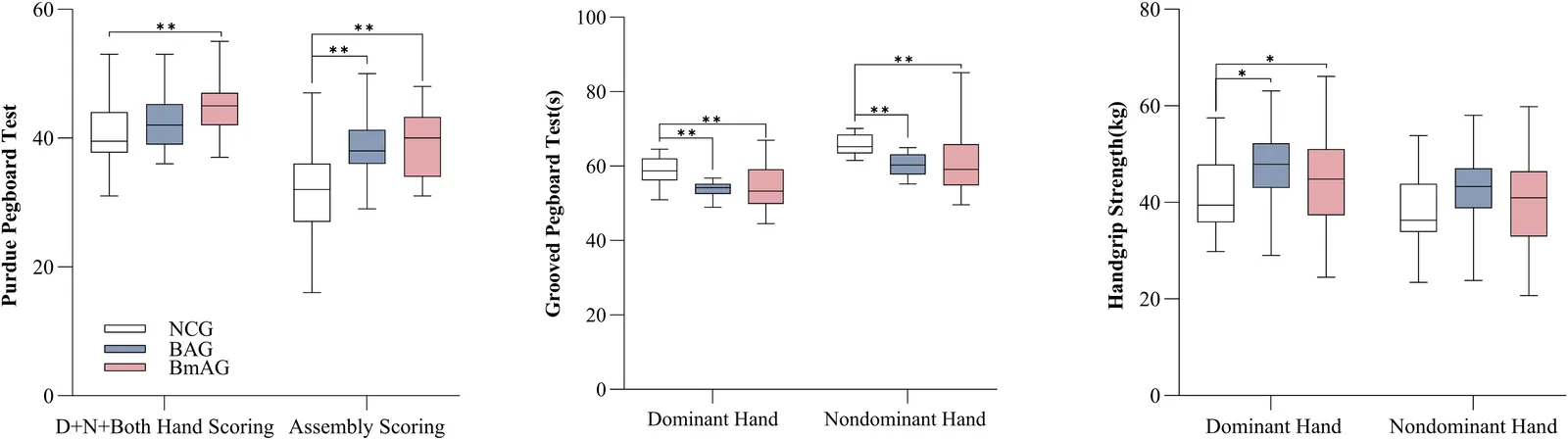
Inter-group differences were analyzed by one-way ANOVA with post hoc Tukey test. GPT, Grooved Pegboard Test; NCG, Non-athlete control group; BAG, Basketball athletes group; BmAG, Badminton athletes group; D, dominant; N, non-dominant; D+N+Both Hands, sum of dominant, non-dominant and both hands. *P < 0.05, **P < 0.01 vs. NCG; For variables with unequal variances, Welch’s ANOVA with Games–Howell post hoc tests was used instead (see Results for details).

## Results

### Purdue pegboard test

All PPT variables were normally distributed and satisfied the assumption of homogeneity of variances (all P > 0.05). Therefore, between-group differences were examined using one-way analysis of variance (ANOVA), followed by Tukey’s HSD *post hoc* comparisons. For the dominant-hand task, the mean scores were 14.76 ± 1.89, 15.86 ± 1.54, and 16.43 ± 1.56 for the NCG, BAG, and BmAG, respectively. A significant overall group effect was observed (F = 7.432, P = 0.001, η² = 0.146). *Post hoc* comparisons showed that both the BAG and BmAG scored significantly higher than the NCG (BAG vs. NCG: P = 0.037, d = 0.63; BmAG vs. NCG: P < 0.001, d = 0.96). For the non-dominant-hand task, the corresponding scores were 13.76 ± 1.90, 14.43 ± 1.87, and 14.96 ± 1.72 for the NCG, BAG, and BmAG, respectively. The overall group effect was significant (F = 3.107, P = 0.049, η² = 0.067). *Post hoc* analysis showed that the BmAG scored significantly higher than the NCG (P = 0.038, d = 0.66), whereas the other pairwise comparisons were not significant. For the bimanual task, the mean scores were 11.56 ± 1.47, 11.96 ± 1.62, and 13.00 ± 1.52 for the NCG, BAG, and BmAG, respectively. A significant group effect was observed (F = 6.661, P = 0.002, η² = 0.133). The BmAG scored significantly higher than both the NCG and BAG (BmAG vs. NCG: P = 0.002, d = 0.96; BmAG vs. BAG: P = 0.033, d = 0.66), whereas no significant difference was found between the BAG and NCG (P = 0.587, d = 0.25). For the combined dominant-hand, non-dominant-hand, and bimanual score, the mean values were 40.10 ± 5.27, 42.26 ± 2.53, and 44.40 ± 4.81 for the NCG, BAG, and BmAG, respectively. The overall group effect was significant (F = 5.738, P = 0.005, η² = 0.117). *Post hoc* analysis showed that the BmAG scored significantly higher than the NCG (P < 0.001, d = 1.08). For the assembly task, the mean scores were 32.30 ± 7.36, 38.30 ± 4.30, and 39.10 ± 4.70 for the NCG, BAG, and BmAG, respectively. A significant between-group difference was observed (F = 12.610, P < 0.001, η² = 0.225). Both the BAG and BmAG scored significantly higher than the NCG (BAG vs. NCG: P < 0.001, d = 0.99; BmAG vs. NCG: P < 0.001, d = 1.10), whereas no significant difference was found between the BAG and BmAG (P = 0.863, d = 0.17). After Holm–Bonferroni correction across the 16 outcome-level omnibus tests, the group effects for the dominant-hand, bimanual, combined, and assembly tasks remained significant, whereas the non-dominant-hand effect (P = 0.049) did not remain significant and is interpreted as exploratory. For the combined score, the BAG did not differ significantly from the NCG (P > 0.05). The pairwise BmAG–BAG difference in the bimanual task (P = 0.033) was not part of the omnibus correction family and would likewise not remain significant under a multiplicity-adjusted threshold; it is therefore interpreted with corresponding caution.

### Grooved pegboard test

GPT performance was assessed using task completion time (**Figure 1B**). For the dominant-hand task, the data were normally distributed but did not satisfy the assumption of homogeneity of variances. Therefore, Welch’s ANOVA was used. A significant group effect was observed, with completion times of 59.51 ± 3.14 s, 53.92 ± 1.87 s, and 54.22 ± 5.90 s for the NCG, BAG, and BmAG, respectively (F = 14.730, P < 0.001, η² = 0.291). Both the BAG and BmAG completed the task significantly faster than the NCG (BAG vs. NCG: P < 0.001, d = 2.01; BmAG vs. NCG: P < 0.001, d = 1.24). No significant difference was observed between the BAG and BmAG (P = 0.991, d = 0.06). For the non-dominant-hand task, the data were also normally distributed but showed unequal variances. Welch’s ANOVA revealed a significant group effect. The mean completion times were 65.86 ± 2.58 s, 60.32 ± 2.71 s, and 61.16 ± 8.64 s for the NCG, BAG, and BmAG, respectively (F = 8.740, P < 0.001, η² = 0.167). The BAG and BmAG completed the task significantly faster than the NCG (BAG vs. NCG: P < 0.001, d = 2.09; BmAG vs. NCG: P = 0.004, d = 0.73). The difference between the BAG and BmAG was not significant (P = 0.827, d = 0.13). Both GPT group effects remained significant after Holm–Bonferroni correction.

### Handgrip strength test

The handgrip strength results are presented in **Table 3** and **Figure 1C**. For dominant-hand grip strength, the data were normally distributed but showed unequal variances; therefore, Welch’s ANOVA was performed. The mean values were 41.53 ± 7.24 kg, 48.08 ± 7.31 kg, and 44.18 ± 9.61 kg for the NCG, BAG, and BmAG, respectively. A significant group effect was observed (F = 4.831, P = 0.010, η² = 0.100). The BAG demonstrated significantly greater grip strength than the NCG (P = 0.007, d = 0.90), whereas the BmAG did not differ significantly from either the NCG (P = 0.427, d = 0.31) or the BAG (P = 0.162, d = 0.45). For non-dominant-hand grip strength, the mean values were 38.32 ± 6.81 kg, 43.05 ± 7.23 kg, and 39.31 ± 8.88 kg for the NCG, BAG, and BmAG, respectively. The overall group effect did not reach statistical significance (F = 3.040, P = 0.053, η² = 0.065). Although the BAG showed greater grip strength than the NCG in the pairwise comparison (P = 0.013, d = 0.66), this result should be interpreted cautiously because the omnibus test was not statistically significant. No significant differences were observed between the BmAG and either the NCG or BAG. After Holm–Bonferroni correction, the dominant-hand grip-strength effect (P = 0.010) did not remain significant and is therefore interpreted as exploratory; the non-dominant-hand effect was not significant in either analysis.

### Cognitive function tests

The cognitive test results are presented in **Table 4**. For the MoCA, Welch’s ANOVA revealed a significant group effect. The mean scores were 27.07 ± 1.53, 28.60 ± 1.10, and 28.53 ± 1.83 for the NCG, BAG, and BmAG, respectively (F = 10.549, P < 0.001, η² = 0.183). Both the BAG and BmAG scored significantly higher than the NCG (BAG vs. NCG: P < 0.001, d = 1.15; BmAG vs. NCG: P = 0.001, d = 0.87). No significant difference was found between the BAG and BmAG (P = 0.865, d = 0.04). For the SWR condition, the mean scores were 104.86 ± 15.40, 115.13 ± 19.92, and 114.20 ± 14.38 for the NCG, BAG, and BmAG, respectively. A significant group effect was observed (F = 3.305, P = 0.041, η² = 0.071). *Post hoc* analysis showed that both the BAG and BmAG performed significantly better than the NCG (BAG vs. NCG: P = 0.033, d = 0.57; BmAG vs. NCG: P = 0.021, d = 0.62). No significant between-group difference was found for SWR error counts. The mean values were 1.30 ± 1.44, 0.93 ± 1.12, and 1.36 ± 1.25 for the NCG, BAG, and BmAG, respectively (F = 0.966, P = 0.385, η² = 0.022). For the Stroop Color Naming (SCN) condition, the mean scores were 72.53 ± 10.49, 75.73 ± 9.74, and 73.40 ± 8.60 for the NCG, BAG, and BmAG, respectively. No significant group effect was observed (F = 0.845, P = 0.429, η² = 0.019). Similarly, SCN error counts did not differ significantly among groups. The mean values were 1.56 ± 1.49, 1.53 ± 1.33, and 1.53 ± 1.35 for the NCG, BAG, and BmAG, respectively (F = 0.005, P = 0.995, η² = 0.000). For the SCW condition, the mean scores were 47.06 ± 11.86, 48.66 ± 8.47, and 45.63 ± 7.62 for the NCG, BAG, and BmAG, respectively. No significant between-group difference was observed (F = 0.740, P = 0.480, η² = 0.017). SCW error counts also did not differ significantly among groups, with mean values of 2.13 ± 1.80, 2.10 ± 1.32, and 2.23 ± 1.58 for the NCG, BAG, and BmAG, respectively (F = 0.056, P = 0.946, η² = 0.001). After Holm–Bonferroni correction, the MoCA group effect remained significant, whereas the SWR effect (P = 0.041) did not remain significant and is interpreted as exploratory.

**Table 4:** Cognitive function assessment.

Parameter		NCG (n=30)	BAG (n=30)	BmAG (n=30)	Overall F	P/η²
MoCA		27.07 ± 1.53	28.60 ± 1.10*	28.53 ± 1.83*	10.549	<0.001/0.183
SCWT	SWR	104.86 ± 15.4	115.13 ± 19.92*	114.20 ± 14.38*	3.305	0.041/0.071
	SWR Error Count	1.30 ± 1.44	0.93 ± 1.12	1.36 ± 1.25	0.966	0.385/0.022
	SCN	72.53 ± 10.49	75.73 ± 9.74	73.40 ± 8.60	0.845	0.429/0.019
	SCN Error Count	1.56 ± 1.49	1.53 ± 1.33	1.53 ± 1.35	0.005	0.995/0.000
	SCW	47.06 ± 11.86	48.66 ± 8.47	45.63 ± 7.62	0.740	0.480/0.017
	SCW Error Count	2.13 ± 1.80	2.10 ± 1.32	2.23 ± 1.58	0.056	0.946/0.001

Inter-group differences were analyzed by one-way ANOVA with post hoc Tukey test. MoCA, Montreal Cognitive Assessment; SCWT, Stroop Color Word Test; SWR, Stroop Word Reading; SCN, Stroop Color Naming; SCW, Stroop Color-Word; NCG, Non-athlete control group; BAG, Basketball athletes group; BmAG, Badminton athletes group. *P < 0.05, **P < 0.01 vs. NCG; no symbol indicates P > 0.05. For the MoCA, which showed unequal variances, Welch’s ANOVA with Games–Howell post hoc tests was used; all SCWT variables were analyzed by standard one-way ANOVA with Tukey’s HSD. No comparison between the two athlete groups (BAG vs. BmAG) reached statistical significance in this table.

## Discussion

Previous studies have often compared athletes and non-athletes as broad categories, which may obscure differences in movement structure, technical demands, and perceptual–cognitive requirements across sports. The present cross-sectional study extended this general comparison by examining associations between long-term participation in basketball and badminton and hand dexterity, handgrip strength, and cognitive performance. Overall, both athlete groups performed better than non-athletes on selected measures of manual dexterity and global cognitive screening; however, the pattern of performance differed between sports. Badminton athletes demonstrated superior performance in the bimanual Purdue Pegboard task, whereas basketball athletes showed greater dominant-hand grip strength than non-athletes. Both athlete groups also completed the Grooved Pegboard tasks more rapidly than non-athletes and obtained higher MoCA scores. These findings suggest that basketball and badminton participation is associated with shared perceptual–cognitive–motor characteristics as well as sport-specific patterns of hand function. However, because the present study used a cross-sectional design, these findings should not be interpreted as evidence that sport training directly caused the observed advantages. It is also possible that individuals with naturally superior manual dexterity, grip strength, cognitive characteristics, or motivation were more likely to select and persist in these sports. In addition, because the study did not include an active comparison group from lower-strategy or closed-skill sports (e.g., swimming, athletics, or resistance training), the athlete–control differences may partly reflect general physical activity and overall athletic status rather than the specific strategic demands of basketball and badminton. The outcomes that did not survive multiplicity correction (non-dominant-hand pegboard performance, dominant-hand grip strength, and Stroop word reading) temper the evidence for fully distinct sport-specific profiles; the central claim is accordingly framed in terms of the robust, correction-surviving differences in bimanual dexterity, assembly performance, pegboard completion time, and global cognition, with the remaining contrasts acknowledged as exploratory.

The Purdue Pegboard Test showed that both basketball and badminton athletes scored higher than non-athletes on the dominant-hand task. For the non-dominant-hand task, badminton athletes scored higher than non-athletes, whereas basketball athletes did not differ significantly from either comparison group. These findings indicate that the association between sport participation and manual dexterity was not uniform across hands or sports. The results are broadly consistent with research suggesting that sport expertise is associated with advantages in selected laboratory-based motor and cognitive tasks involving processing speed, response selection, and action organization (Voss et al., 2010). Mann et al. similarly proposed that skilled athletes may demonstrate more efficient perceptual cognitive processing, which could facilitate visual information pickup, anticipation, and continuous motor planning (Mann et al., 2007).

The bimanual Purdue Pegboard task revealed a more specific sport-related pattern. Badminton athletes outperformed both basketball athletes and non-athletes, whereas basketball athletes did not differ significantly from non-athletes. The assembly task showed a related but less sport-specific pattern, with both athlete groups scoring higher than non-athletes. Together, these findings suggest that badminton participation is associated with better performance on tasks requiring coordinated use of both hands and the organization of multiple fine-motor actions. Nevertheless, this pattern should be interpreted as an association rather than a direct consequence of badminton training. One possible explanation is that the non-dominant hand in badminton may serve an active role in trunk stabilization, balance regulation, postural orientation, stroke preparation, and movement recovery. Biomechanical analyses of racket sports indicate that the non-dominant arm contributes to counter-rotation, balance, and stroke coordination during high-velocity racket swings (Bahamonde and Knudson, 2003). Badminton also requires rapid adjustment of the racket arm while the contralateral side contributes to whole-body alignment and dynamic balance. By contrast, basketball also requires bilateral hand use, but many key technical actions may place greater emphasis on dominant-hand control, particularly during dribbling, passing, ball recovery, and finishing under dynamic and contact-related conditions (Drinkwater et al., 2008; Stojanović et al., 2018). Therefore, the superior bimanual performance observed in badminton athletes may reflect the combined influence of sport-specific coordination demands and pre-existing individual characteristics that contributed to sport selection or continued participation. Previous work has also suggested that badminton performance relies on rapid and precise coordination of multiple body segments and effective upper-limb control (Chen et al., 2022; Phomsoupha and Laffaye, 2015). However, because the present study did not directly assess sport-specific movement patterns or pre-training abilities, the proposed explanation remains tentative.

The Grooved Pegboard Test further indicated a shared manual-performance advantage among the two athlete groups. Both basketball and badminton athletes completed the dominant- and non-dominant-hand tasks faster than non-athletes, while no significant difference was observed between the two athlete groups. The Grooved Pegboard requires peg insertion into oriented grooves and therefore involves visual discrimination, visuomotor matching, forearm pronation–supination, hand-posture adjustment, and rapid corrective control (Merker and Podell, 2011). The faster performance of both athlete groups may be associated with the common demands of interactive sports, including rapid information processing, continuous action adjustment, and close perception–action coupling. Mann et al. emphasized that skilled athletes often demonstrate advantages in predictive tracking, rapid decision-making, and error correction (Mann et al., 2007), whereas Williams et al. described visual perception and motor execution in sport as components of a tightly coupled functional system (Williams et al., 1999). From this perspective, the shared advantage of basketball and badminton athletes in the Grooved Pegboard Test may reflect common characteristics related to perception–cognition–action integration. However, these results cannot determine whether such characteristics were developed through sport participation or whether individuals who already possessed stronger visuomotor abilities were more likely to participate in these sports. The absence of a difference between the two athlete groups also suggests that some aspects of manual dexterity may be associated with general athletic experience or regular physical activity rather than with the specific characteristics of basketball or badminton.

More pronounced sport-related differences were observed for handgrip strength. Basketball athletes demonstrated greater dominant-hand grip strength than non-athletes, whereas badminton athletes did not differ significantly from either group. For non-dominant-hand grip strength, the overall group effect did not reach statistical significance. Although the pairwise comparison suggested greater grip strength in basketball athletes than in non-athletes, this result should be interpreted cautiously because the omnibus test was not statistically significant. Thus, the most consistent grip-strength finding was the association between basketball participation and greater dominant-hand grip strength. This pattern is compatible with the functional demands of basketball. Basketball performance involves ball handling, dribbling, passing, catching, ball recovery, and finishing, often during rapid directional changes and physical interaction. These activities require athletes to control the ball while maintaining postural stability and coordinating the upper and lower limbs under dynamic conditions (Drinkwater et al., 2008; Stojanović et al., 2018). Individuals who participate in basketball may therefore develop, or may already possess, greater dominant-hand force capacity. However, because grip strength was not assessed before sport participation and no longitudinal data were available, it cannot be concluded that basketball training caused the observed difference.

In contrast, badminton racket handling places substantial emphasis on rapid acceleration, precise timing, flexible control, and finely graded force modulation. Phomsoupha and Laffaye highlighted the importance of visual tracking, rapid racket-head acceleration, and distal control precision in badminton performance (Phomsoupha and Laffaye, 2015). These requirements may place less emphasis on maximal static grip force than on rapid transfer and regulation of force across the wrist, forearm, shoulder, trunk, and lower limbs. Cronin et al. similarly noted that the relationship between grip strength and sport performance is strongly task dependent (Cronin et al., 2017). Therefore, the absence of a clear grip-strength advantage in badminton athletes should not be interpreted as evidence of poorer hand function. Rather, it may indicate that badminton participation is associated with a performance profile emphasizing rapid force modulation, racket control, and coordination instead of maximal static force production. The dissociation between manual dexterity and handgrip strength is important. Badminton athletes showed an advantage in bimanual dexterity without demonstrating a clear advantage in maximal grip strength, whereas basketball athletes showed greater dominant-hand grip strength but did not outperform badminton athletes on the bimanual task. These findings support the view that hand function is multidimensional and that different sports may be associated with different combinations of force capacity, coordination, visuomotor control, and bilateral motor organization.

Both basketball and badminton athletes obtained higher MoCA scores than non-athletes. This result may indicate an association between participation in these sports and higher performance on a brief measure of global cognitive status. Previous research has linked sport expertise with attentional control, information-processing speed, and executive function (Voss et al., 2010), and high-level sport performance requires the rapid extraction, integration, and use of complex environmental information (Mann et al., 2007).Basketball and badminton both involve continuous monitoring of the environment, anticipation of an opponent’s actions, response selection, and rapid adjustment of technical and tactical behavior. However, the interpretation of the MoCA findings requires considerable caution. The MoCA was originally developed primarily as a screening instrument for mild cognitive impairment and other clinical cognitive disorders, rather than as a sensitive measure of subtle executive-function differences among healthy young adults. The participants in the present study were healthy university athletes and non-athletes aged approximately 18–22 years, and their mean MoCA scores were relatively high. In the present sample, 62.2% of participants scored ≥ 28 points and 27.8% obtained the maximum score of 30 (NCG: 30.0% ≥ 28; BAG: 83.3%; BmAG: 73.3%), indicating that the score distribution was compressed toward the upper limit. The limited between-group variation and the generally high scores may therefore reflect a ceiling effect. In this context, the higher scores observed in the athlete groups should not be interpreted as evidence of broad or clinically meaningful cognitive superiority. The MoCA was included as a brief, standardized measure of general cognitive status and to provide a broad characterization of the sample; it was not intended to provide a comprehensive assessment of sport-related executive-function adaptations. Future studies should use cognitive instruments designed for high-functioning young adults, including more sensitive measures of inhibitory control, working memory, attentional shifting, processing speed, anticipation, and decision-making.

The SCWT results also support a cautious interpretation. The athlete groups performed better than the non-athlete group in the Stroop Word Reading condition, but no significant group differences were observed for SCN, SCW, or error counts. Thus, the present data do not support a generalized advantage across all measured domains of inhibitory control and cognitive flexibility. The word-reading difference may reflect faster reading or processing speed rather than a broad enhancement of executive control. Two explanations may account for the absence of differences in the other SCWT conditions. First, the healthy young participants may have performed near the upper range of the test, thereby reducing its sensitivity to subtle group differences. Second, any cognitive characteristics associated with basketball or badminton may be task specific. They may be more apparent in perceptual motor integration, anticipation, dynamic decision-making, and sport-relevant response selection than in relatively abstract and decontextualized laboratory measures of inhibitory control. In addition, academic achievement and educational experience—which were not assessed in the present study may influence performance on general cognitive screening and verbal tasks. Therefore, the higher MoCA and word-reading scores should be interpreted as group-associated differences in selected cognitive measures, not as evidence of a direct or generalized cognitive benefit caused by sport participation.

From a motor-control perspective, hand function should be understood as an emergent property of the entire action system rather than as an isolated peripheral ability. Dynamic systems theory and constraints-led approaches propose that skilled movement emerges through self-organization under the interacting constraints imposed by the individual, task, and environment (Davids et al., 2007; Newell, 1986). Manual performance reflects coordinated interactions among postural control, visual guidance, bilateral coordination, proximal stability, and distal upper-limb execution. Basketball and badminton impose different combinations of these constraints. Basketball emphasizes forceful whole-body coordination during locomotion, rapid directional changes, ball control, and physical contact. Badminton places greater demands on rapid and precise movement, asymmetrical coordination, anticipatory control, and racket manipulation within a constrained spatial and temporal environment. The present dissociation between greater dominant-hand grip strength in basketball athletes and superior bimanual coordination in badminton athletes is compatible with this constraints-based framework. Nevertheless, the findings cannot establish that these sport-specific characteristics were produced by training. They may also reflect the self-selection of participants whose pre-existing motor abilities were compatible with the demands of a particular sport. The bimanual advantage observed in badminton athletes may reflect the development—or pre-existing presence—of a non-dominant limb that contributes to postural stability, spatial orientation, rhythm regulation, and movement preparation. In basketball, the non-dominant hand is also important, but it may more frequently provide supportive or assistive control while the dominant hand performs the primary ball-handling action. Similarly, the dominant-hand grip-strength advantage in basketball athletes may be associated with the repeated coupling of hand-force production with trunk stability, lower-limb propulsion, ball protection, and contact-based actions. In badminton, racket performance may depend more strongly on rapid multijoint velocity transfer and precise regulation of force than on maximal static grip strength (Phomsoupha and Laffaye, 2015; Cronin et al., 2017). Bimanual coordination may also involve cognitive control and distributed neural networks in addition to peripheral motor output (Serrien et al., 2007; Swinnen, 2002). This perspective may help explain why the badminton advantage was particularly apparent in the bimanual Purdue Pegboard task but not in dominant-hand grip strength. However, no direct neural, kinematic, or muscle-activation measures were collected. Consequently, these proposed perceptual, neural, and motor-control mechanisms remain theoretically informed interpretations rather than mechanisms directly demonstrated by the current data.

### Limitations and future directions

Several limitations should be acknowledged. First, the cross-sectional design precludes causal inference; the observed differences may reflect pre-existing characteristics rather than training effects. Longitudinal and intervention studies are needed to clarify this. Second, the MoCA was designed as a clinical screening tool and may have shown ceiling effects in this healthy young sample. Future research should employ more sensitive cognitive measures for high-functioning young adults. Third, several confounding variables—including academic achievement, background physical activity, resistance-training history, hand size and other manual anthropometric characteristics, sleep, nutrition, and additional lifestyle factors—were not assessed. Future studies should control for these through matched designs or multivariable analyses. Fourth, the absence of non-interactive sport controls (e.g., swimming, track and field) limits conclusions about whether differences reflect strategic complexity or general athletic status. Fifth, the modest, predominantly male sample (6 women, 84 men) restricts generalizability and precluded sex-stratified analyses or sex-adjusted models. Future work should recruit larger, more diverse samples. Finally, general laboratory measures may lack ecological validity for sport-specific demands. Future studies should integrate sport-specific behavioral testing with neurophysiological, electromyographic, and kinematic methods. In addition, the Stroop conditions were administered in a fixed order, which constitutes a potential practice and fatigue confound, and the athlete groups were recruited from a shared club environment, and participants recruited from the same club teams may be statistically clustered, which would violate the independence assumption underlying the one-way ANOVAs; residual club-level clustering therefore cannot be fully excluded.

## Conclusion

This cross-sectional study found that basketball and badminton athletes performed better than non-athletes on selected measures of manual dexterity and brief global cognitive screening. Both athlete groups completed the Grooved Pegboard tasks faster and obtained higher MoCA scores. Badminton athletes showed superior bimanual Purdue Pegboard performance, whereas basketball athletes demonstrated greater dominant-hand grip strength than non-athletes. These findings indicate associations between sport participation and shared perceptual–cognitive–motor characteristics, together with sport-related differences in hand function. However, they do not establish that training caused these advantages, as pre-existing differences in motor ability, cognition, motivation, or other unmeasured factors may have influenced sport selection and participation. The MoCA results also require caution because it was designed primarily for clinical screening and may have shown a ceiling effect in this healthy young sample. The findings provide preliminary implications for sport-specific assessment, but require prospective validation. Future studies should include additional sport groups, more sensitive cognitive measures, and assessments of academic achievement, physical activity, resistance-training history, sleep, nutrition, and other lifestyle factors.

## Data Availability

The raw data supporting the conclusions of this article will be made available by the authors, without undue reservation.
